# Soil Fertility, Phosphorus Fractions, and Maize Yield as Affected by Poultry Manure and Single Superphosphate

**DOI:** 10.1155/2015/616213

**Published:** 2015-02-10

**Authors:** A. O. Ojo, M. T. Adetunji, K. A. Okeleye, C. O. Adejuyigbe

**Affiliations:** ^1^Institute of Agricultural Research and Training, Obafemi Awolowo University, P.M.B 5029, Moor Plantation, Ibadan, Oyo State, Nigeria; ^2^Federal University of Agriculture, Abeokuta, Nigeria

## Abstract

A field experiment was conducted in 2007 and 2008 on a slightly acidic alfisol. Poultry manure (PM) was applied at 0, 5 t ha^−1^, 10 t ha^−1^, 15 t ha^−1^, and 20 t ha^−1^ in combination with SSP at 0, 15 kg P ha^−1^, 30 kg P ha^−1^, 45 kg P ha^−1^, and 60 kg P ha^−1^, which was replicated three times. The pH and organic C were significantly increased by the application of PM alone while available P was highly increased by the sole application of SSP. Plant tissue P was significantly increased with the application of 30 kg P ha^−1^ while the largest grain yield was obtained when PM at 20 t ha^−1^ was combined with SSP at 60 kg P ha^−1^. The buildup of organic P was observed when PM was applied at 15 t ha^−1^ while the combination of the two treatments increased residual P and Fe-P. However, P occlusion was effectively reduced with the sole application of PM. Organic P and residual P however had a strong positive relationship with the grain yield. Comparing the sole and combined application of the treatments, the combined application was more effective for most of the parameters observed.

## 1. Introduction

Phosphorus (P) is one of the essential elements for plant growth and the number of plants available P in the soil is often inadequate to meet plant requirements. Phosphorus is a macronutrient that plays a number of important roles in plant. Adequate phosphorus results in higher grain production, improved crop quality, greater stalk strength, increased root growth, and earlier crop maturity. Mokwunye et al. [1] and Warren [2] stated that phosphorus deficiency is one of the largest constraints to food production in tropical African soils due to low native P and high fixation by iron and aluminum oxides. It has also been established that phosphorus is relatively unavailable for plant uptake in highly weathered soils like the Ultisols [3]. In order to meet the need of phosphorus in these soils, farmers make use of inorganic fertilizers for their crops. However, P fertilizer is not readily available to these farmers because of scarcity in some cases and also due to the high cost of these fertilizers. Recently, farmers especially in the developed countries make use of organic manure which has been found to be effective like inorganic fertilizers for the release of some nutrients especially phosphorus but the resultant yield has been found not to be as high as when inorganic fertilizer is combined with the organic manure. Combining organic manure with P fertilizers has been found to not only increase yield but also reduce P fixation and subsequently increase phosphorus availability and uptake [3]. The study however aims at studying the effect of combined application of poultry manure and single superphosphate on phosphorus forms, soil fertility, and the yield maize.

## 2. Materials and Methods

The field experiment was conducted at I.A.R&T experimental field at Ibadan in 2007 and 2008 early planting season (see Table 1). The experimental design was a 5 × 5 factorial experiment in a RCBD replicated three times. The plot size was 5 m × 4 m (20 m^2^) with spacing of 75 cm × 50 cm to give a total plant population of 106,666 plants ha^−1^. Three seeds of maize (TZB-SR) were planted after 2 weeks of treatment incorporation. The seedlings were thinned to two per stand two weeks after planting. The plots were not tilled in the early season (May) for the second year (2008) in order to avoid mixing up of treatments. Gramoxone and primextra were used as herbicides. Weeding was done at the 4th and 8th week after planting.

Leaf sampling of maize was done at eight weeks after planting. Leaf just below and opposite the ear was sampled randomly per plot. Ears of maize were allowed to dry on the stalk. Ears in the four middle rows of each plot were harvested (making allowance for two guard rows) on each side of the plot. The cobs were dehusked and weighed. Ten cobs per plots were randomly selected for cob weight measurement. The cobs were sun dried and the grains were weighed. Yield per hectare was computed at 12% moisture content for maize. Soil samples were analyzed before and after for phosphorus fractions and soil nutrient composition.

### 2.1. Soil Physical and Chemical Analysis

Particle size distribution was determined by the hydrometer method [4] using sodium hexametaphosphate as the dispersing agent. Soil pH was determined in distilled water (1 : 1 soil water ratio) with a pH meter. Exchangeable cations (K, Na, Ca, and Mg) by extraction, with ammonium acetate (pH = 7). Available P was extracted with 0.03 N NH_4_F in 0.025 N HCl solution [5] and P in the extract was analysed colorimetrically by the molybdenum blue method at 660 nm. Soil organic carbon was determined by wet oxidation with sulphuric acid [6]. Total P was determined by wet digestion using perchloric acid and nitric acid while organic P was by ignition method. Phosphorus concentration in the digests was determined using the ascorbic acid method.

### 2.2. Phosphorus Fractionation Study

Fractionation procedure by Chang and Jackson [7] was used to quantify different forms of inorganic P in the studied soils (Table 2). Fractionation was done on the soil before and after amendment for incubation, greenhouse, and field experiment. The sequence of the chemical extraction and a brief description of the targeted P forms are shown in Table 2.

### 2.3. Determination of Phosphorus in the Extracts

The phosphorus in the extracts was determined by the methyl blue colour method of Murphy and Riley [8]. An aliquot of 10 mL was used in developing the colour. The determination of P in NH_4_F, NaOH, and DCB extracts however requires the following pretreatment before the development of the colour.

- NH_4_F extract: 0.05 g of H_3_BO_3_ per 50 mL of the extract was added before taking the aliquot.
- NaOH extract: after precipitating the organic matter with conc. H_2_SO_4_, the pH of the extract was adjusted with NaOH using 2,4-dinitrophenol indicator. An aliquot of the clear solution was then taken into a 50 mL volumetric flask.
- DCB extract (reductant soluble P): 10 mL of the extract was pipetted into a test tube and 1 mL of HClO_4_ was added to it and boiled to destroy the dithionite in the extract. It was then transferred into a 50 mL volumetric flask and 3 mL of 5% ammonium molybdate solution was added while distilled water was used to make up to mark. Absorbance was read after 30 minutes.

### 2.4. Analysis of Poultry Manure Sample

Total analysis of the sample was done by weighing 0.5 g of poultry manure, adding 10 mL of HNO_3_/HClO_4_, (2 : 1), and digesting at 150°C. The temperature was increased at 1.5 hours to 230°C and 2 mL of HCl/H_2_O (1 : 1) was added. The digestion was now continued for another 30 minutes [9].

### 2.5. Statistical Analysis

The data collected were subjected to analysis of variance using the statistical analysis system (SAS)—general linear model [10]. Means were separated by Duncan multiple range test.

## 3. Results

### 3.1. Initial Chemical Properties of the Soil Used

The pH of the soil was slightly acidic and therefore a need for the application of organic manure. The soil was low in N, Na, and organic carbon while P was moderately available in it. It was however adequate with cations like Ca, Mg, and K (Table 3).

### 3.2. Characterization of the Poultry Manure Used and the Initial Phosphorus Fractions of the Soil

Nitrogen in the poultry manure used was 5.82% which was similar to the result observed for phosphorus (5.54%) while potassium was low in the soil (0.94%). Organic C had a high percentage of 13.38% which led to the resultant C/N ratio of 2.3 (Table 4). Also notable was the result obtained for Fe (1555 mg/kg).

The order of abundance of the phosphorus fractions before the application of treatments was organic P > Fe-P ≡ occluded P > residual P (Table 5).

### 3.3. Effect of Combined Application of Poultry Manure and SSP on the Soil Chemical Properties

There was a decrease in pH as a result of the sole application of SSP in 2007, which further decreased in 2008 (Table 6). Application of poultry manure alone increased the pH of the soil significantly when compared to the other treatments. However, the combined application of poultry manure and SSP increased the pH of the soil in 2007 with a further increase in 2008. In other words, poultry manure alone can be compared favorably with the combined application of poultry manure and SSP in increasing soil pH especially in slightly acidic soil. Effect of SSP on organic C was not significant and decreased when compared to the initial value. However, the application of poultry manure increased organic C significantly when poultry manure was applied alone in 2007. In 2008, organic C further increased with the sole application of poultry manure. The effect of the combined application of poultry manure and SSP was not significant on organic carbon in 2007 but significant in 2008 with the combined application of poultry manure and SSP. Available P increased over the control either with the sole application of poultry manure or SSP or when the two treatments were combined. However, in 2007 the sole application of SSP was more effective in increasing available P than the sole application of poultry manure but was the most effective with the combined application of poultry manure and SSP, while, in 2008, the combined treatments were not significant but the sole application was.

### 3.4. Effect of the Combined Application of Poultry Manure and SSP on Plant Tissue P Concentration and Grain Yield of Maize

Effect of poultry manure and SSP alone with their combinations was significant in both years (Table 7). In 2007, plant tissue P was high when the treatments were applied solely but combined application was highly significant. Plant tissue P continued to increase in 2008 with the sole and combined application but with the most significant increase with the sole application of SSP.

The sole application of SSP had no significant effect on the grain yield of maize in 2007 while the sole application of poultry manure and the combined application of the two treatments were significant in the same year. However, in 2007, the highest grain yield of maize was obtained by the sole application of poultry manure. In 2008, effect of the sole application of poultry manure and SSP on maize grain yield was significant while the combined application was not. The combined application of the two treatments gave the highest grain yield in 2008.

### 3.5. Effect of the Combined Application of Poultry Manure and SSP on Phosphorus Fractions

Effect of the sole and combined application of poultry manure and SSP was significant in both 2007 and 2008 (Table 8). In 2007, organic P was significantly increased over the initial value when poultry manure was applied solely than when SSP was applied alone. Combining the two treatments also increased organic P significantly in 2007. The application of poultry manure alone however gave the highest increase in organic P in 2007. In 2008, organic P reduced with the sole application of SSP but increased when poultry manure was applied alone and with some combined treatments. The sole application of poultry manure was again effective in 2008 in increasing organic P. Residual P increased over the control when poultry manure and SSP were applied alone and when combined in both years. Residual P increased in 2007 with the combined application of the two treatments but reduced in 2008. A similar trend to that of residual P was observed for Fe-P but with few exceptions when the 2 treatments were combined. In 2008, the largest buildup of Fe-P was observed with the combined application of poultry manure and SSP. In both years of cropping, sole application of poultry manure was effective in reducing P occlusion more than the combined application.

### 3.6. Relationship among the P Fractions, Soil Chemical Properties, and Maize Grain Yield

In 2007, the pH of the soil had a strong negative relationship with Al-P and occluded P but positive relationship with residual P (Table 9), while in 2008, increase in the pH of the soil favored the buildup of Fe-P (Table 10). Available P in 2007 had a positive correlation with organic P, Ca-P, and Fe-P but a negative correlation with occluded P. Organic C had no significant relationship with the P fractions in 2007 but a positive correlation with organic P and a negative correlation with occluded P in 2008. Organic P and residual P contributed positively to the maize grain yield in both 2007 and 2008.

## 4. Discussion

### 4.1. Initial Chemical Properties of the Soil Used

The pH of the soil was slightly acidic and this will make Fe-P be more available than the other inorganic P fractions. Although phosphorus was moderately available, poultry manure and SSP were further applied in order to study the effect of continuous application of organic and inorganic fertilizer on such soils yearly as has been practiced by many farmers in Nigeria.

### 4.2. Characterization of the Poultry Manure Used and Initial Phosphorus Fractions of the Soil

A common and traditional practice has utilized manure as an organic fertilizer or soil improver due to its contribution of organic matter and nutrients, mainly N and P [11]. The high percentage of nitrogen in the poultry manure used could have been due to the decomposition of poultry manure during the curing stage before application to the soil with a subsequent release of N which was detected during the analysis. Manure in past studies has been applied according to the nitrogen needs of the soil [12]. Macronutrients such as phosphorus are part of the advantage of using organic waste in agriculture, which may be recovered and incorporated into natural cycle and this was evident in the percentage of phosphorus in the poultry manure used. It also contains a high level of organic matter which generates changes in chemical, physical, and biological soil amendment properties [12].

Organic P was the highest P fraction at the initial stage of this study. About two-thirds of the phosphorus in fresh manure is in organic form [12]. The organic P pool is usually considered a major source of organic phosphorus and can be converted to inorganic P by ignition or wet digestion. Initial abundance of Fe-P could be attributed to the acidic nature of the soil; however occluded P also had the same value which could mean there is an equilibrium reaction between the labile and the nonlabile P fractions.

### 4.3. Effect of Combined Application of Poultry Manure and SSP on the Soil Chemical Properties

Acidity in sols if not properly managed will eventually make the soil be low or not yielding and therefore a need for amendment. In this study, application of poultry manure compared favourably with the combined application of poultry manure and SSP in increasing the pH of the soil while applying inorganic fertilizer alone decreased the soil pH. Soil pH of an acidic Ultisol in the eastern Nigeria was observed to increase soil pH [13]. Sole and combined organic and inorganic fertilizer has been found to increase soil nutrients favorably. The combined application of the two treatments was highly effective in increasing soil organic C in both 2007 and 2008. Earlier studies on poultry manure ability to increase soil organic C have been reported [13, 14], likewise when it is combined with SSP [15]. Single superphosphate was however more effective than the sole application of poultry manure for increasing the available P content of the soil although the combined application of the two treatments still gave the highest value [16]. The effectiveness of SSP for available P over poultry manure could be due to the immediate release of phosphate into the soil when SSP is being applied.

### 4.4. Effect of the Combined Application of Poultry Manure and SSP on Plant Tissue P Concentration and Grain Yield of Maize

High plant tissue P was obtained in 2007 and 2008 with the sole and combined application of poultry manure and SSP. However, the sole application of SSP was however sustainable in the 2nd year to cause a further increase in the concentration of P in the shoot. A similar result has been observed by Adeniyan and Ojeniyi, [17]. The increase in the plant tissue P in 2008 was as a result of P accumulation observed in both years, which would have resulted in the transfer to the shoot of the plant instead of being washed into nearby river and hereby preventing eutrophication.

When the treatments were applied solely, application of poultry manure alone was more effective than SSP, that is, considering the high grain yield. Although this was in the first year of planting, the combined application of poultry manure and SSP was again sustainable in the second year. The combined application of organic and inorganic resources has been found to favor maize production [14].

### 4.5. Effect of the Combined Application of Poultry Manure and SSP on Phosphorus Fractions

The buildup of organic P was observed in both years of this study with the sole application of poultry manure and this signifies the importance of organic manure when considering amendments for an acidic soil. Addition of soybean residue and wheat residue or in combination with fertilizer P favored the buildup of organic P in a study [18]. The inorganic P fractions considered, that is, residual P and Fe-P, was significantly increased when poultry manure was combined with SSP. This indicates the ability of the combined application of poultry manure and SSP to maintain the fertility of the soil through increase in the labile P fraction [19]. Residual P however reduced in 2008 and this was also observed by Agbenin and Goladi, [20] in an experiment involving the addition of P fertilizers or in combination with cow manure. Significant was the ability of poultry manure alone to reduce P occlusion in both years, that is, occluded P, which further confirms an earlier study which states that addition of organic materials to soil decreases P fixation [3]. Inorganic and organic products are generated during the partial decomposition of organic waste and organic acids can be adsorbed into the soil surface thereby decreasing the potential P adsorption by blocking the formation of complexes [21].

### 4.6. Relationship among the P Fractions, Soil Chemical Properties, and Maize Grain Yield

Organic P and Fe-P contributed significantly to the increase observed in available P in 2007 while occluded P decreased with an increase in available P. This further confirms the importance of poultry manure while considering soil amelioration. In both years of cropping, organic P was sustainable in increasing grain yield but Fe-P was only effective in 2007. Residual P (a nonlabile P fraction) equally contributed to the increase observed in grain yield in both years. In other words, there would have been an equilibrium reaction between the nonlabile P (residual P) and labile P (Fe-P) fraction which would have subsequently resulted in an increase in available P.

## 5. Conclusion

Application of poultry manure alone was effective in increasing the pH, organic C, and available P content of the soil. However, application of SSP was highly significant in increasing the available P content of the soil and this was evident in the increase obtained for the plant tissue P in 2008, while, for grain yield, the most effective was the combined application of poultry manure and SSP. Increase in organic P fraction was as a result of the application of poultry manure while the combined application of poultry manure and SSP increased the Fe-P fraction. Poultry manure alone was also the most effective treatment for the reduction of P occlusion in the soil. Significant were also the importance of poultry manure and the combined application of poultry manure and SSP exhibited through the strong relationship that existed among available P, Fe-P, and organic P. Although poultry manure was compared favorably with the combined application of poultry manure and SSP, the combination of the treatments was more suitable for the sustainability of the fertility of the slightly acidic soil.

## Figures and Tables

**Table 1:** Source of the soil used.

Location	State	Coordinates	Vegetation	Soil series	Soil class (USDA)
Ibadan	Oyo	Lat. 7°30′ Long. 3°54′	Rainforest	Iwo	Ferrudulf

**Table 2:** Sequential P fractionation procedures and targeted P forms.

Location	Extractants	Equilibration	Washing	Targeted
NH_4_Cl-P	IM NH_4_Cl	30 mins	None	Saloid-bound P (loosely bound P)

NH_4_F-P	0.5 M NH_4_F	1 hour	None	Al-P

NaOH-P	0.1 M NaOH saturated Nacl + 5 drops of conc. H_2_SO_4_	15 mins	Saturated Nacl	Fe-P

Sodium + citrate Sodium dithionite P	0.3 M sodium citrate + 1 g solid Sodium dithionate + sat Nacl	Shake 15 mins, preheat 15 mins at 85°C after sodium citrate, additional 15 mins after dithionite addition	Saturated Nacl	Reductant-soluble P

NaOH-P	0.1 M NaOH + few drops of conc. H_2_SO_4_ to remove colour	15 mins	Saturated NaCl	Occluded P

H_2_SO_4_-P	0.25 M H_2_SO_4_ + 25 mL Sat. Nacl	1 hour	Saturated Nacl	Ca-P

Residual P	Conc. HNO_3_ + HCl + 30% H_2_O_2_	Variable until complete	None	Organically stable organic and inorganic P

**Table 3:** Chemical and physical properties of the soil used for the study.

Parameters	
pH (H_2_O)	6.4
O.M (g kg^−1^)	24.08
N (g kg^−1^)	0.2
P (mg kg^−1^)	14.2
Exchangeable cations (c molc kg^−1^)	
Ca	1.14
Mg	0.82
K	0.47
Na	0.09
Particle size (g kg^−1^)	
Sand	740
Clay	120
Silt	140

**Table 4:** Characterization of the poultry manure used.

pH	7.03
Nitrogen (N) (%)	5.82
Phosphorus (P) (%)	5.54
Potassium (K) (%)	0.94
Na (%)	1.3
SO_4_-S (%)	0.14
Organic C (%)	13.34
C/N	2.3
Calcium (%)	8.04
Magnesium (%)	0.61
Iron (mg kg^−1^)	1555
Copper (mg kg^−1^)	33.3
Zinc (mg kg^−1^)	100.3
Manganese (mg kg^−1^)	180

**Table 5:** Phosphorus fractions of the soil before planting (mg kg^−1^).

Phosphorus fractions (mg kg^−1^)	
Total P	272.84
Organic P	45.90
Fe-P	33.39
Occluded P	33.39
Residual P	16.16

**Table 6:** Effect of poultry manure (PM) and SSP on some soil chemical properties in 2007 and 2008.

Treatments		pH (2007)	pH (2008)	Organic C, % (2007)	Organic C, % (2008)	Available P, mg kg^−1^ (2007)	Available P, mg kg^−1^ (2008)
PM (t ha^−1^)	SSP (kg P ha^−1^)						
0	0	6.23	6.30	0.19	0.16	5.90	6.07
	15	6.73	6.73	0.62	0.85	92.57	96.78
	30	6.32	6.30	1.02	1.27	39.41	39.91
	45	6.51	6.51	1.26	0.74	69.60	70.02
	60	6.42	6.87	0.75	0.89	42.56	41.18

5	0	7.06	7.06	0.65	1.05	41.50	41.70
	15	6.53	6.53	0.92	1.90	56.39	56.27
	30	6.52	6.52	1.06	1.42	43.56	43.84
	45	6.65	6.62	1.12	1.29	90.05	87.66
	60	6.92	6.92	0.91	0.97	64.69	65.82

10	0	6.87	6.87	1.03	1.39	64.82	63.30
	15	6.82	6.73	1.03	1.04	59.62	59.74
	30	6.72	6.72	1.37	0.87	67.06	66.74
	45	6.86	6.86	0.98	1.34	83.32	81.65
	60	6.87	6.87	1.02	1.53	60.42	60.42

15	0	6.50	6.50	1.63	1.52	40.05	96.78
	15	6.87	6.93	1.66	2.65	67.71	67.78
	30	6.80	6.80	1.08	1.45	96.82	87.49
	45	6.65	6.72	1.08	1.62	88.31	87.24
	60	6.62	7.16	1.25	1.50	75.57	74.54

20	0	6.93	6.93	1.03	1.75	59.60	53.07
	15	6.60	6.60	0.78	1.01	72.68	76.81
	30	6.60	6.60	1.22	1.26	72.68	79.69
	45	6.50	6.50	0.82	1.12	64.87	64.44
	60	6.97	6.96	1.23	2.60	61.74	94.84

	LSD (*P* ≤ 0.05)						
	SSP	∗	∗	NS	∗	∗	NS
	PM	∗	∗	∗	∗	∗	NS
	SSP × PM	∗	NS	NS	∗	∗	NS

^*^Significant at *P* < 0.05, NS: not significant.

**Table 7:** Effect of poultry manure (PM) and SSP on plant tissue concentration and grain yield in 2007 and 2008.

Treatments		Plant tissue (%) P		Grain yield (t ha^−1^)	
PM (t ha^−1^)	SSP (kg P ha^−1^)	2007	2008	2007	2008
0	0	0.43	0.53	1.33	0.81
	15	0.94	1.04	3.12	3.21
	30	0.82	2.51	2.29	1.45
	45	1.29	2.02	2.57	1.05
	60	0.79	1.93	2.39	1.42

5	0	0.88	0.77	2.76	1.98
	15	1.97	1.56	2.61	1.25
	30	0.87	0.96	2.69	1.67
	45	0.85	2.50	2.71	1.74
	60	0.82	1.24	2.35	2.57

10	0	1.32	0.86	2.87	1.66
	15	1.49	1.82	2.46	2.18
	30	1.01	1.33	2.59	3.02
	45	1.61	0.84	2.48	1.78
	60	1.28	1.35	2.42	3.53

15	0	2.00	1.04	3.63	0.83
	15	1.82	1.01	2.75	3.85
	30	0.87	0.83	2.75	3.50
	45	1.49	1.42	2.60	3.21
	60	0.93	1.02	2.20	3.76

20	0	1.03	0.94	2.82	2.82
	15	0.87	1.36	2.86	2.41
	30	1.36	1.52	2.64	2.44
	45	0.99	2.09	2.36	2.30
	60	0.99	1.35	2.37	3.99

	LSD (*P* ≤ 0.05)				
	SSP	∗	∗	NS	∗
	PM	∗	∗	∗	∗
	SSP × PM	∗	∗	∗	NS

^*^Significant at *P* < 0.05, NS: not significant.

**Table 8:** Effects of poultry manure and SSP on phosphorus fractions in 2007 and 2008.

Treatments		Phosphorus fractions (mg kg^−1^)							
PM (t ha^−1^)	P rates (Kg P ha^−1^)	Organic P 2007	Organic P 2008	Residual P 2007	Residual P 2008	Fe-P 2007	Fe-P 2008	Occluded P 2007	Occluded P 2008
0	0	49.91	50.04	21.83	12.26	38.50	19.48	27.25	28.03
	15	128.96	112.50	36.41	18.55	88.21	21.62	31.61	10.11
	30	135.63	125.02	36.08	17.54	90.24	17.74	22.66	10.53
	45	115.63	140.63	40.73	20.52	68.09	64.92	31.65	11.72
	60	177.50	130.58	54.99	22.97	117.37	18.86	36.72	12.24

5	0	75.03	111.27	47.43	17.45	88.12	50.76	16.61	9.36
	15	138.75	168.75	37.18	15.52	86.82	16.72	19.79	11.08
	30	167.35	146.85	41.47	17.34	88.44	16.85	26.08	9.56
	45	143.14	116.22	48.76	19.31	87.08	137.76	27.82	12.34
	60	100.06	106.83	46.26	19.22	116.29	62.25	21.18	17.14

10	0	105.07	160.05	49.32	17.43	87.15	86.81	18.63	10.52
	15	180.05	118.75	45.24	16.45	88.31	19.07	26.82	8.51
	30	183.73	155.05	51.29	18.63	115.23	15.35	26.18	10.33
	45	160.37	122.50	38.16	21.28	104.62	117.55	44.02	12.88
	60	145.61	130.11	42.28	20.75	117.69	148.94	30.12	11.72

15	0	198.75	112.50	36.61	16.92	61.81	21.62	12.14	17.01
	15	91.85	120.02	45.57	20.07	97.69	18.25	22.89	8.51
	30	124.38	159.38	41.89	18.62	117.68	15.96	38.42	10.64
	45	105.53	174.33	40.11	25.54	98.54	131.93	42.11	14.26
	60	161.86	170.52	34.45	22.76	202.36	152.11	28.95	9.92

20	0	149.35	103.77	47.51	18.91	84.52	16.08	12.46	12.13
	15	99.39	101.88	44.41	16.07	75.13	18.95	32.88	9.37
	30	141.85	174.83	42.39	21.05	117.99	15.97	34.91	11.08
	45	195.00	136.22	54.63	31.62	89.62	13.18	36.81	11.21
	60	171.25	150.02	57.24	23.65	113.12	125.41	39.37	13.21

	LSD (*P* ≤ 0.05)								
	SSP	∗	∗	∗	∗	∗	∗	∗	∗
	PM	∗	∗	∗	∗	∗	∗	∗	∗
	SSP × PM	∗	∗	∗	∗	∗	∗	∗	∗

^*^Significant at *P* < 0.05, NS: not significant.

**Table 9:** Correlation coefficient (*r*) among P fractions, soil chemical properties, and maize grain yield in 2007.

Soil properties	P fractions (mg kg^−1^)			
	Organic P	Fe-P	Occluded P	Residual P
pH	0.098	0.201	−0.550	0.633
Available P	0.514	0.433	−0.528	−0.071
Organic C	0.206	0.115	−0.031	−0.105
Grain yield	0.413	0.145	0.034	0.402

**Table 10:** Correlation coefficient (*r*) among P fractions, soil chemical properties, and maize grain yield in 2008.

Soil properties	P fractions (mg kg^−1^)			
	Organic P	Fe-P	Occluded P	Residual P
pH	0.118	0.458	0.065	−0.144
Available P	0.053	0.173	−0.753	0.048
Organic C	0.500	0.122	−0.557	−0.177
Grain yield	0.403	0.105	0.123	0.412
